# Degradation of bamboo lignocellulose by bamboo snout beetle *Cyrtotrachelus buqueti* in vivo and vitro: efficiency and mechanism

**DOI:** 10.1186/s13068-019-1406-y

**Published:** 2019-04-01

**Authors:** Chaobing Luo, Yuanqiu Li, Ying Chen, Chun Fu, Xiang Nong, Yaojun Yang

**Affiliations:** 1 Bamboo Diseases and Pests Control and Resources Development Key Laboratory of Sichuan Province, Leshan Normal University, No. 778, Riverside Road, Central District, Leshan, 614000 China; 2 College of Food and Biological Engineering, Xihua University, Chengdu, 610039 China

**Keywords:** Bamboo shoots, *Cyrtotrachelus buqueti*, Lignocellulose, Transcriptome, Lignocellulosic enzyme

## Abstract

**Background:**

As an important biomass raw material, the lignocellulose in bamboo is of significant value in energy conversion. The conversion of bamboo lignocellulose into fermentable reducing sugar, i.e. the degradation of bamboo lignocellulose, is an important step in lignocellulose conversion. However, little research has focussed on excavating the enzymes and microbes that are related to the degradation of bamboo lignocellulose, which is important for its utilisation. This study used *Cyrtotrachelus buqueti* (bamboo snout beetle) to evaluate the efficiency of bamboo lignocellulose degradation.

**Results:**

RNA sequencing was conducted to sequence the transcriptome of the insect before and after feeding on bamboo shoots. The expression levels of genes encoding several carbohydrate-active enzymes, such as endoglucanase (*evgtrinloc27093t1* and *evgtrinloc16407t0*) and laccase (*evgtrinloc15173t0* and *evgtrinloc11252t0*), were found to be upregulated after feeding. Faecal component analysis showed that the degradation efficiencies of cellulose, hemicellulose and lignin were 61.82%, 87.65% and 69.05%, respectively. After 6 days of co-culture with crude enzymes in vitro, the degradation efficiencies of cellulose, hemicellulose and lignin in bamboo shoot particles (BSPs) were 24.98%, 37.52% and 26.67%, respectively. These results indicated that lignocellulosic enzymes and related enzymes within the insect itself co-degraded bamboo lignocellulose. These finding can potentially be used for the pre-treatment and enzymatic hydrolysis of bamboo lignocellulose.

**Conclusion:**

Our results showed that intestinal digestive enzymes from *C. buqueti* degraded bamboo shoot lignocellulose both in vivo and in vitro. In addition, the expression levels of many carbohydrate-active enzyme (CAZyme) genes were upregulated in the transcriptome, including those for cellulase, xylanase and ligninase genes. Therefore, we proposed a scheme for applying the lignocellulolytic enzymes from *C. buqueti* to degrade bamboo lignocellulose using genetic, enzymatic and fermentation engineering techniques to overexpress the lignocellulolytic enzymes genes in vitro and obtain large quantities of enzymes that could efficiently degrade bamboo lignocellulose and be used for lignocellulose bioconversion.

**Electronic supplementary material:**

The online version of this article (10.1186/s13068-019-1406-y) contains supplementary material, which is available to authorized users.

## Background

Bamboo is an abundant resource that can be used as a renewable biomass material. It has a short growth cycle, high yield and high lignocellulose content which provide many advantages for bioenergy utilisation [1]. In addition, bamboo requires relatively low amounts of chemicals and nutrients during growth and can be cultivated on marginal land for bio-based products [2]. In total, 500 species of bamboo, from more than 40 genera, can be found throughout China. Bamboo occupies 33,000 km^2^ of the country’s total forested area, which accounts for nearly one-quarter of bamboo forest area worldwide [2]. Moreover, bamboo is a potential feedstock for the production of fuel ethanol and bamboo lignocellulose degradation becomes a hotspot [3–6]. Although many methods for bamboo lignocellulose degradation have been proposed, little research has been devoted to excavate the enzymes related to the degradation of bamboo lignocellulose, which is important for its utilisation. Moreover, most studies have used moso bamboo, whereas only few studies have used *Bambusa emeiensis*, a type of cluster bamboo widely distributed in western China.

Enzymatic hydrolysis is an important step in lignocellulose bioconversion. During this step, cellulose and hemicellulose are converted into their sugar constituents, which can be fermented to produce biofuels [7]. However, the cost and hydrolyzing efficiency of cellulase are quite far removed from large-scale commercial digestion of lignocellulosic feedstock [8]. In nature, there are several good examples of efficient utilisation of plant biomass, called ‘natural biomass utilisation systems’ (NBUSs) [9]. Of these, lignocellulose-feeding insects are most worthy of mentioning, and possess incredible lignocellulose degradation capabilities [10, 11]. In our previous study, we reported that many carbohydrate-active enzyme (CAZyme) genes, which are primarily involved in lignocellulose degradation [12, 13], expressed and lignocellulosic enzyme system existed in the different developmental stages of the bamboo snout beetle *Cyrtotrachelus buqueti* [14]. Nevertheless, some studies have discussed CAZymes obtained from insects, such as termites [15], *Anoplophora glabripennis* (Asian longhorn beetle) [16], *Diacritical virgifera virgifera* (Western corn rootworm) [17], and *Rhynchophorus ferrugineus* (palm weevil) [18]. However, more detailed knowledge about the degradation, like degradation efficiency, mechanism, and so on, still lacked.

In this study, the abilities of adult *C. buqueti* to degrade bamboo lignocellulose in vivo and in vitro were investigated. The digestive systems of *C. buqueti* were extracted before and after feeding for total RNA isolation and high-throughput RNA sequencing. Transcriptional profiling revealed that the expression levels of CAZymes were increased after feeding. The crude enzymes were extracted and used to degrade bamboo shoot particles (BSPs) in vitro over 6 days, and we found that the lignocellulose was partially removed. This indicated that insect enzymes could degrade bamboo shoot lignocellulose both in vivo and in vitro, which could provide new enzyme resources for the biodegradation of bamboo lignocellulose.

## Results and discussion

### Transcriptome sequencing and assembly

Clean reads were obtained from raw reads after removing reads that were of low quality or were ambiguous; adapters and duplicates were also removed. This resulted in 72,931,350 and 70,126,886 clean reads in the control and treatment *C. buqueti* libraries, respectively. All clean reads were assembled into transcripts using Trinity RNA-Seq [19]. The resulting 108,854 transcripts ranged in length from 201 to 13,018 nucleotides. After assembling these reads into unigenes and discarding unigenes that were less than 200 nucleotides long, 22,776 unigenes remained. These unigenes ranged in length from 201 to 12,666 nucleotides, with N50 unigenes having a length of 2040 nt (Additional file 1: Table S1).

### Taxonomic analysis of assembled unigenes

The assembled unigenes were annotated against several databases: non-redundant (nr) (14,212), conserved domains (CDD) (10,562), Swiss-Prot (11,077), protein families (PFAM) (9547), gene ontology (GO) (12,362), the Kyoto encyclopaedia of genes and genomes (KEGG) (8778), Translation from EMBL (TrEMBL) (14,274) and euKaryotic Orthologous Groups (KOG) (9088) (Additional file 1: Table S1). After functional annotation, the numbers of sequences from five different species matching the insect unigenes were calculated from the annotation characteristics. As displayed in Additional file 2: Table S2, the five species were *Dendroctonus ponderosae* (mountain pine beetle) (52.1%), *Tribolium castaneum* (red flour beetle) (25.9%), *Oryctes borbonicus* (scarab beetle) (1.9%), *Homo sapiens* (1.2%), and *Lasius niger* (0.6%) representing 82% of all annotated species.

### Functional annotation results and differentially expressed genes

**Fig. 1:**
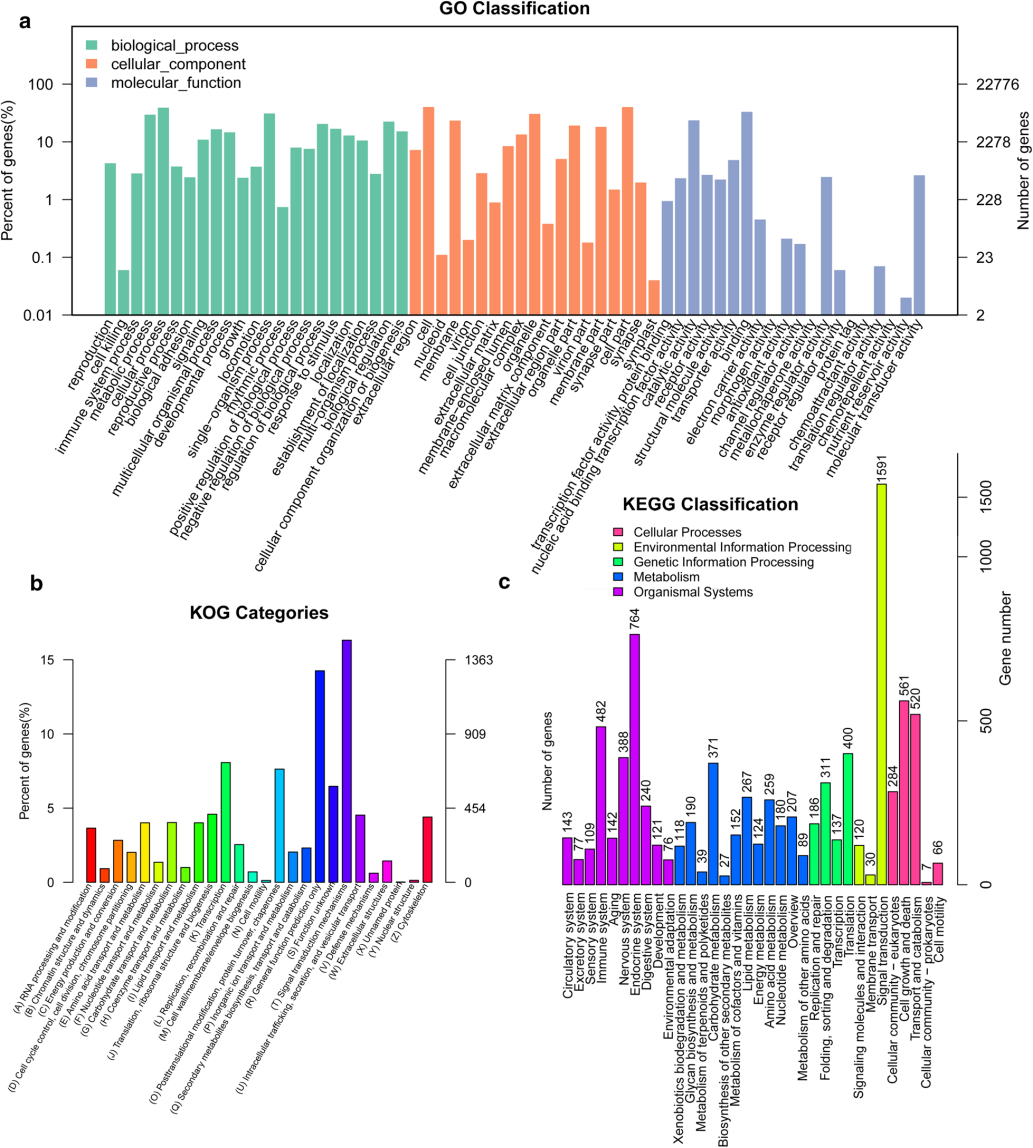
Histogram of GO (**a**), KOG (**b**) and KEGG (**c**) unigene classifications

Of the unigenes, 12,362 were annotated into 62 sub-categories belonging to the following three main GO categories: biological process (BP), cellular component (CC) and molecular function (MF) (Fig. 1a). There were 23 BP sub-categories, 19 CC sub-categories and 20 MF sub-categories. The top ten sub-categories were cell (9110 unigenes), cell part (9085 unigenes), cellular process (8863 unigenes), binding (7533 unigenes), single-organism process (7062 unigenes), organelle (6930 unigenes), metabolic process (6720 unigenes), catalytic activity (5358 unigenes), membrane (5308 unigenes) and biological regulation (5104 unigenes). KOG classification placed 9088 unigenes into 26 functional categories (Fig. 1b). Signal transduction mechanisms was the largest cluster (1483 unigenes), followed by general function prediction (1296 unigenes) and transcription (733 unigenes). The top three categories had 38.6% of unigenes annotated to the KOG database. In all, 8778 unigenes were classified into the following five KEGG functional categories (Fig. 1c): cellular process (1438 unigenes; 16.38% of which were annotated to the KEGG database), environmental information processing (1741; 19.83%), genetic information processing (1034; 11.78%), metabolism (2023; 23.05%) and organismal system (2542; 28.96%). The top three sub-categories were endocrine system, translation and cell growth and death.

**Fig. 2:**
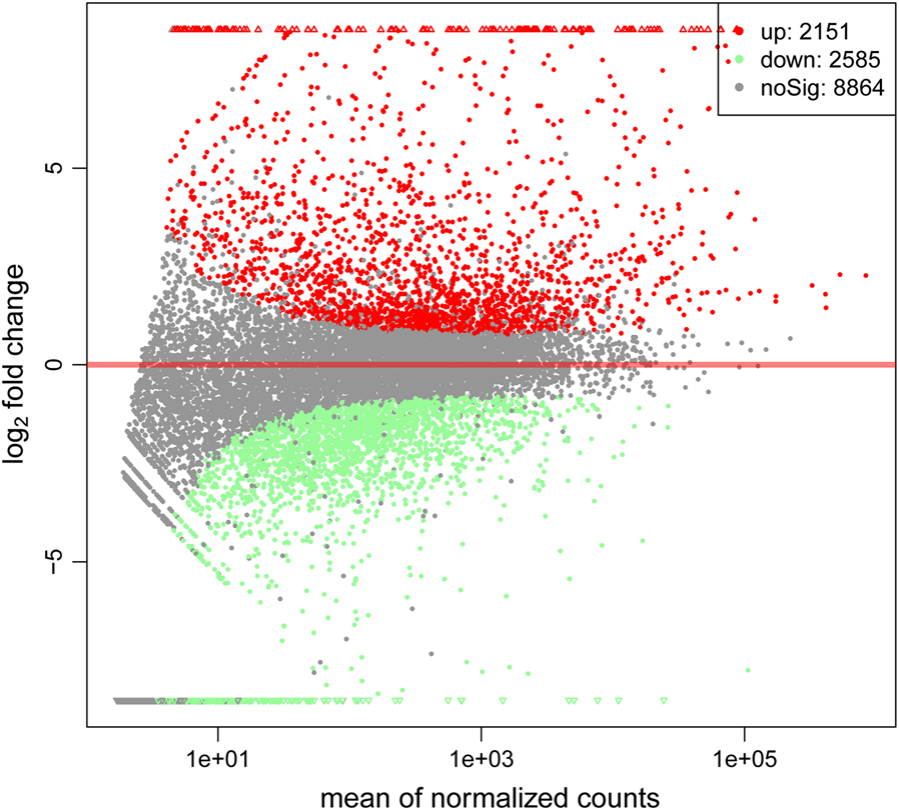
Volcano plot of differentially expressed genes between fed and unfed insects

Among the genes identified, 3337 were differentially expressed with 1512 upregulated and 1825 downregulated between the control and the treated groups, using a false discovery rate ≤ 0.05 and a fold change ≥ 2 as significant cut-offs (Fig. 2).

### Identification of carbohydrate-active enzymes

The main enzyme related to lignocellulose degradation was CAZyme, which can be divided into the following six main categories: GHs, glycosyltransferases (GTs), PLs, CEs, AAs and CBMs [7]. The functions of GHs, GTs, CEs, PLs and CBMs were related mainly to lignocellulosic polysaccharide degradation, whereas the AAs played a more important role in lignin depolymerisation [8]. We subsequently conducted a search to find all CAZyme genes in the transcriptome. We predicted the total proteins of the *C. buqueti* transcriptome using the *e*-value = 1e^−5^. The results indicated that 511 unigenes contained multiple domains that were assigned to CAZyme families as follows: 46 GH families (130 GHs), 53 GT families (184 GTs), 8 CE families (63 CEs), 1 PL family (1 PL), 7 AA families (52 AAs) and 14 CBM families (81 CBMs) (Additional file 3: Figure S1).

The GH families were primarily represented by the families GH1, GH13, GH18, GH2, GH20, GH28, GH31, GH5, GH45, GH47 and GH48. Of these proteins, 11 candidate proteins were identified from the transcriptome as GH1 (Additional file 4: Table S3; Additional file 5: Table S3; Additional file 6: Table S4), which putatively exhibits, among others, β-glucosidase and β-galactosidase activity [14]. Two proteins were assigned to GH45, which exhibits endoglucanase activity [16]. The CAZymes include more than 40 GH45 cellulases, including the activities of different enzymes, such as β-mannosidase (EC 3.2.1.25), chitosanase (EC 3.2.1.132), endo-β-1,4-glucanase (endocellulase, EC 3.2.1.4) and others [20]. Eleven candidates were assigned to GH5, which exhibits endoglucanase activity [21]. Two proteins were assigned to GH48, which is considered a subfamily of the GH5 class of enzymes and acts mainly as a cellulase [21]. One protein was assigned to GH9, which shares substrate specificities with GH5 and GH45 because of possible convergent evolution in terms of enzymatic function [22]. Eight proteins were assigned to GH28, with endo-polygalacturonase activity playing a key role in pectin degradation [23]. Six proteins were assigned to GH16, another group of enzymes with xyloglucan xyloglucosyltransferase activities [24] and encoding β-1,3-glucanases [25].

AAs play an important role in lignin degradation [8]. In total, 52 AA proteins were identified from the transcriptome, comprising 5 laccases (Lac) (AA1), 2 manganese peroxidase (MnPs) (AA2), 31 glucose-methanol-choline (GMC) oxidoreductases (aryl alcohol oxidases and vanillyl-alcohol oxidases; AA3 and AA4, respectively) and other AAs (AA5, AA7 and AA8) (Additional file 4: Table S3; Additional file 5: Table S3; Additional file 6: Table S4). Members of the GMC oxidoreductase superfamily were believed to provide hydrogen peroxide for lignin peroxidase (LiP) and MnP to participate in lignin degradation [26]. These results indicated that many CAZyme family genes exist in the insect intestine, which may give it the ability to degrade lignocellulose.

### Expression profile of carbohydrate-active enzymes

**Fig. 3:**
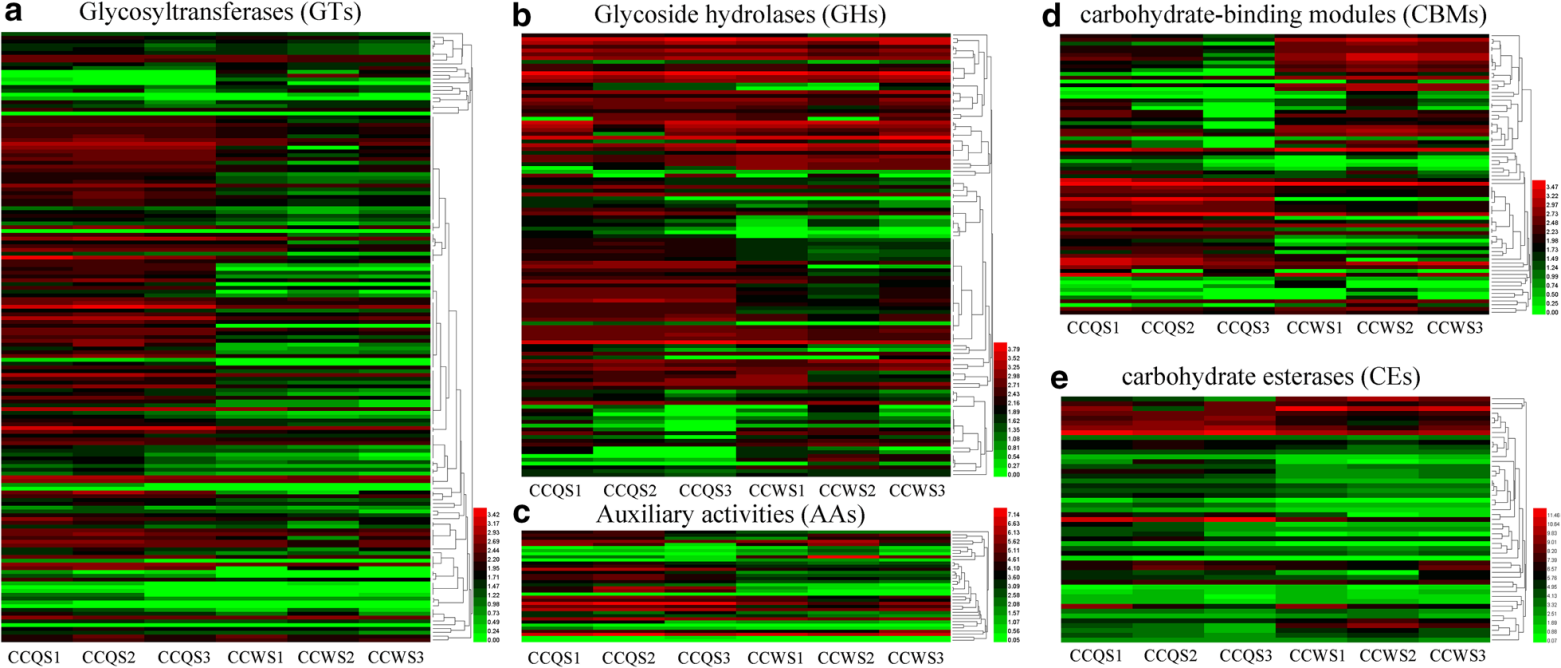
Hierarchical clustering of the expression of CAZyme family genes. **a** GTs, **b** GHs, **c** AAs, **d** CBMs and **e** CEs. GTs: glycosyltransferases, GHs, glycoside hydrolases; CEs, carbohydrate esterases; CBMs, carbohydrate-binding domains; AAs, auxiliary activities; CAZymes, carbohydrate-active enzymes. The black area represents no expression level

We summarised the expression patterns of all CAZyme genes in the transcriptome. The genes that were not expressed in most samples were deleted, and 439 genes (117 GHs, 161 GTs, 74 CBMs, 51 CEs and 36 AAs) remained for analysis. A cluster analysis of the CAZyme gene transcripts revealed that the expression patterns of GH, GT, CE, AA and CBM were divided mainly into two categories: one with no obvious difference between the control and treatment groups and the other in which the expression in the treatment group was higher than that in the control group (Fig. 3). Specifically, for the GHs, among the 117 expressed GH genes, ten unigenes (*evgtrinloc27093t1*, *evgtrinloc16407t0*, *evgtrinloc18622t0*, *evgtrinloc1398t1*, *evgtrinloc18224t0*, *evgtrinloc23175t1*, *evgtrinloc30289t0*, *evgtrinloc6297t0*, *evgtrinloc21178t0* and *evgtrinloc9801t0*) were highly expressed in the treatment group and the top two GH transcripts (*evgtrinloc27093t1* and *evgtrinloc16407t0*) belonged to GH5 (Fig. 3b), which exhibits endoglucanase activity [25]. Among the AAs, seven unigenes (*evgtrinloc13823t0*, *evgtrinloc44713t0*, *evgtrinloc19848t0*, *evgtrinloc13145t0*, *evgtrinloc21246t0*, *evgtrinloc6364t0* and *evgtrinloc15876t1*) were highly expressed in the treatment group and the top two transcripts (*evgtrinloc13823t0* and *evgtrinloc44713t0*) belonged to AA3 (Fig. 3c), which is believed to provide hydrogen peroxide for LiP and MnP to participate in lignin degradation [30]. Among the CEs, eight unigenes (*evgtrinloc23745t0*, *evgtrinloc13466t0*, *evgtrinloc10926t0*, *evgtrinloc24946t0*, *evgtrinloc4951t1*, *evgtrinloc10708t0*, *evgtrinloc21604t1* and *evgtrinloc5945t0*) were highly expressed in the treatment group and the top transcript (*evgtrinloc23745t0*) belonged to CE10 (Fig. 3e), which exhibits carboxylesterase and xylanase activities as well as mannosidase, galactosidase, xyloglucosyltransferase and acetylxylan esterase activities involved in hemicellulose degradation [27].

### Gene expression analysis of lignocellulolytic enzyme-encoding genes after feeding

**Fig. 4:**
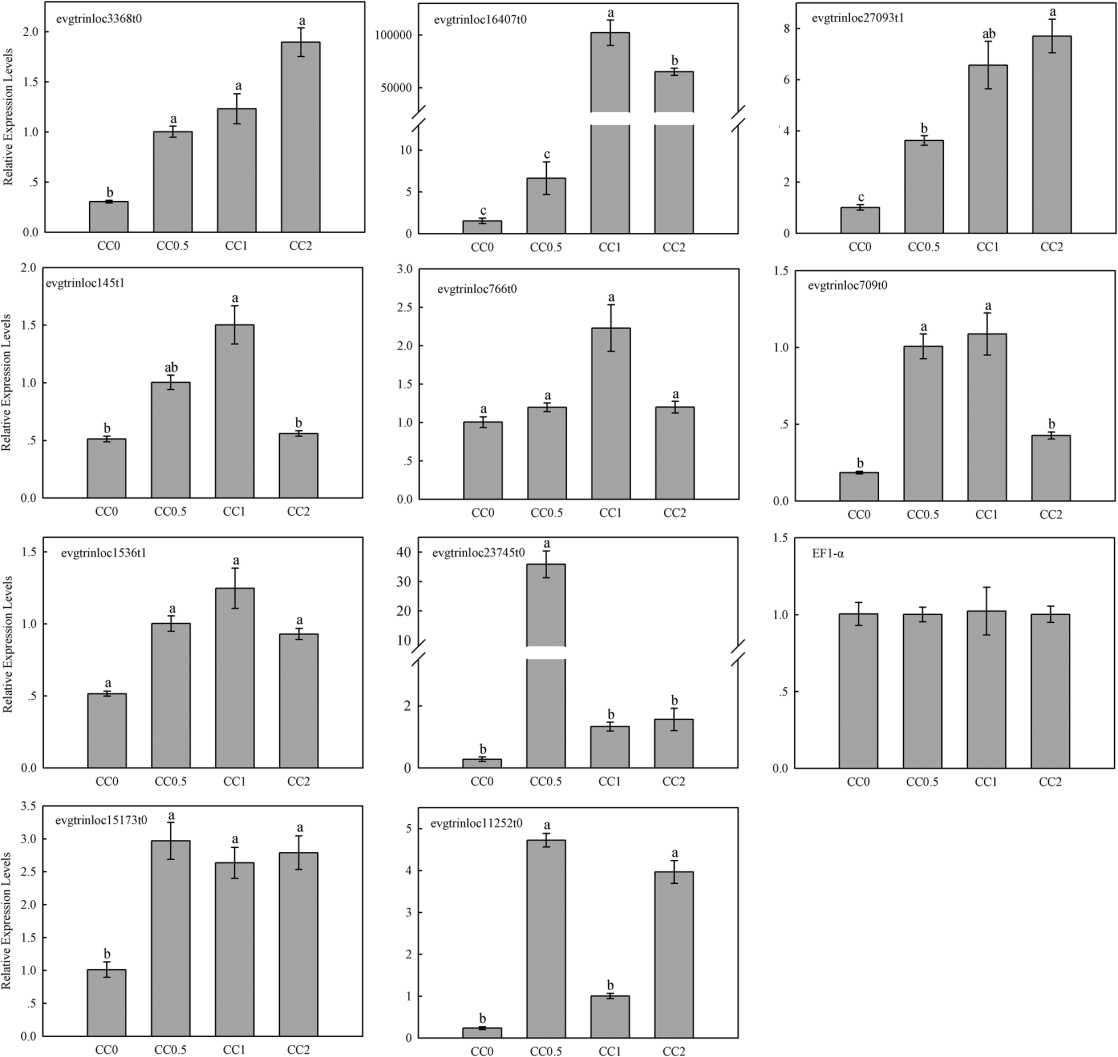
Quantitative real-time polymerase chain reaction (qRT-PCR) analysis of the expression of ten candidate genes in the intestine of *Cyrtotrachelus buqueti* at different time points after feeding. The different normal letters indicate a significant difference in gene expression at different time points (*p* = 0.05; n = 3)

To provide some insight into the expression patterns of lignocellulolytic enzyme-encoding genes in the gut of *C. buqueti*, qRT-PCR was conducted on the following ten genes: three endoglucanase genes (*evgtrinloc3368t0*, *evgtrinloc27093t1* and *evgtrinloc16407t0*), two β-glucosidase genes (*evgtrinloc709t0* and *evgtrinloc1536t1*), two exoglucanase genes (*evgtrinloc145t1* and *evgtrinloc766t0*), one CE10 gene (*evgtrinloc23745t0*) and two laccase genes (*evgtrinloc15173t0* and *evgtrinloc11252t0*), using the primers described in Additional file 9: Table S6, with *EF1*-*ɑ* acting as the reference gene. The expression of each of these ten genes in the gut at 0, 0.5, 1 and 2 h after feeding was determined. *Evgtrinloc3368t0*, *evgtrinloc27093t1*, *evgtrinloc16407t0*, *evgtrinloc145t1* and *evgtrinloc766t0* were upregulated after feeding, while *evgtrinloc23745t0*, *evgtrinloc1536t1*, *evgtrinloc709t0*, *evgtrinloc15173t0* and *evgtrinloc11252t0* were upregulated initially and then decreased after feeding (Fig. 4). This suggested that the lignocellulolytic enzyme-encoding genes were upregulated after insect feeding, which was in accordance with the RNA sequencing.

### Comparison of insect enzymes activities after feeding on bamboo shoots

**Fig. 5:**
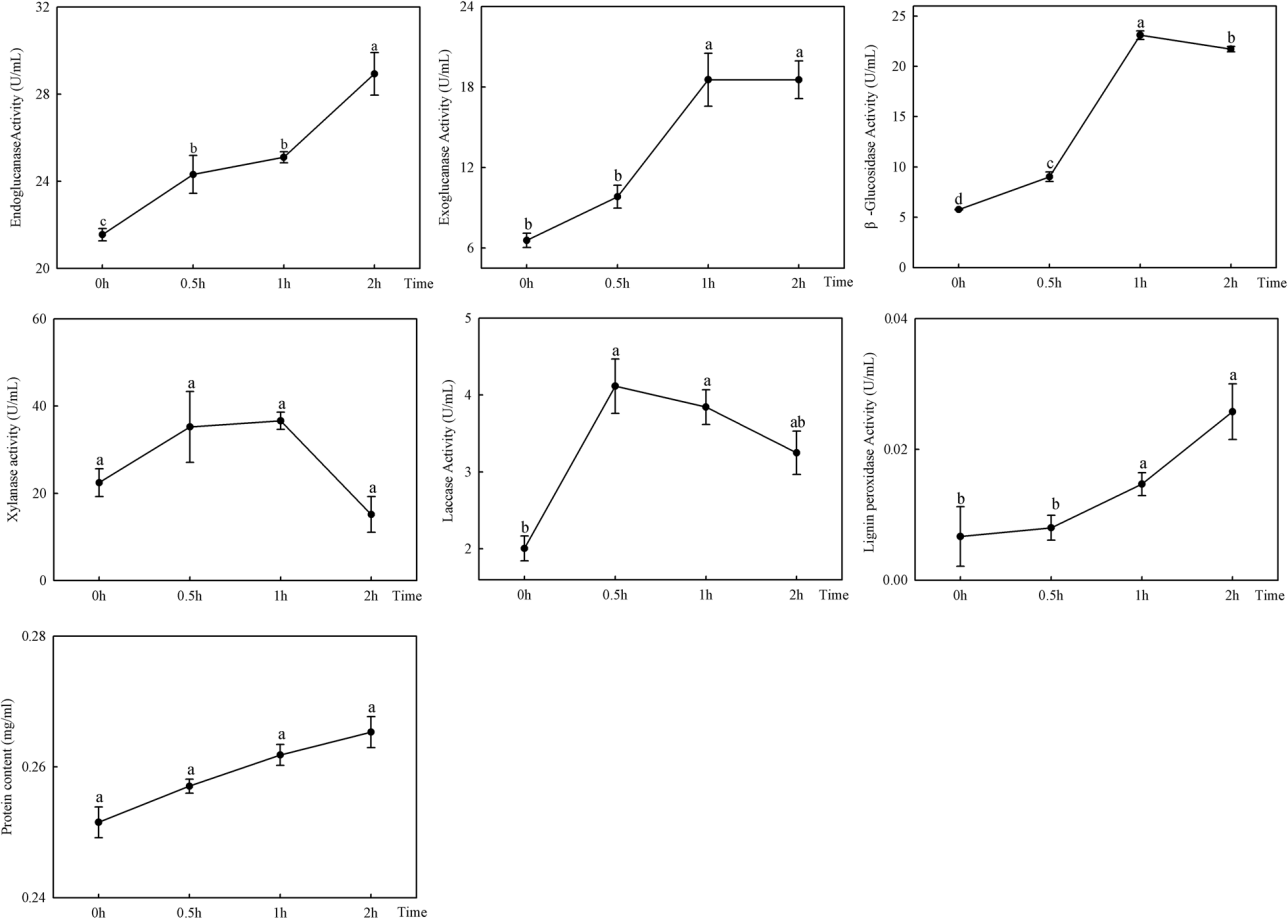
Lignocellulolytic enzyme activities in *Cyrtotrachelus buqueti* intestines at different time points after feeding. The different normal letters indicate a significant difference in enzyme activities at different time points (*p* = 0.05; n = 5)

We detected the lignocellulolytic enzyme activities of endoglucanase, β-glucosidase, xylanase-like enzyme, exoglucanase, Lac and LiP in *C. buqueti* at 0, 0.5, 1 and 2 h after feeding to further confirm the insect’s degradative ability. As presented in Fig. 5, lignin peroxidase activity increased, while laccase activity initially increased and then decreased (Fig. 5). MnP, Lac and LiP are considered to be the three most important enzymes in lignin degradation [28]. We found that the activity of endoglucanase increased, and β-glucosidase, xylanase-like enzyme and exoglucanase exhibited maximum activities at 1 h after feeding and then decreased (Fig. 5). In general, cellulose degradation is attributed to the synergistic action of three classes of GHs, endoglucanases, exoglucanases and β-glucosidases [29], and xylanases are needed to completely degrade hemicellulose polysaccharides [30].

### Scanning electron microscopy (SEM) of bamboo shoots in the intestine of *C. buqueti* after feeding

**Fig. 6:**
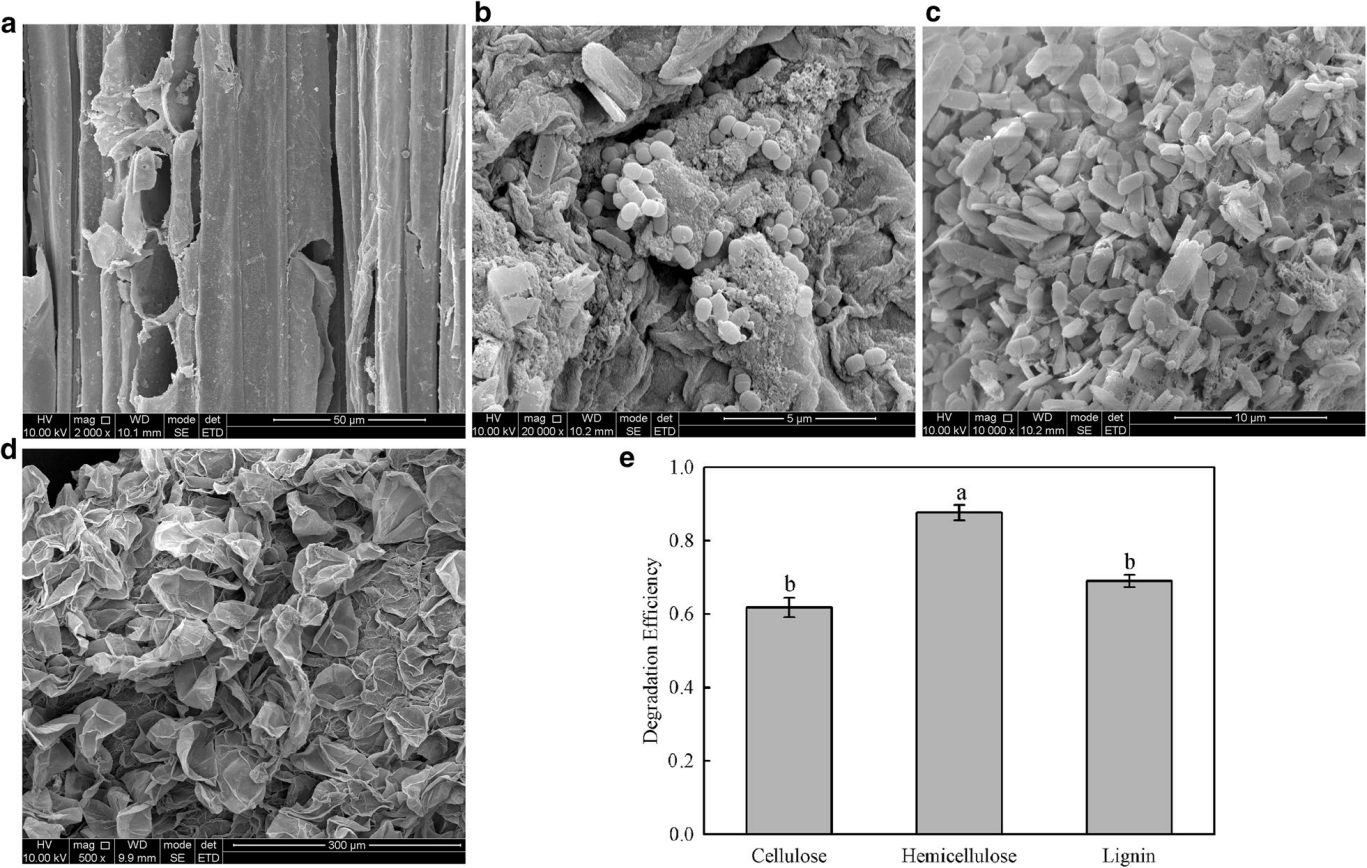
Scanning electron microscopy (SEM) of bamboo shoot particles (BSPs) and degradation of BSPs in faecal materials of *Cyrtotrachelus buqueti*. **a** SEM for BSPs obtained from bamboo shoots; **b** SEM for BSPs obtained from the insect’s foregut; **c** SEM for BSPs obtained from the insect’s midgut; **d** SEM for BSPs obtained from the insect’s hindgut; **e** degradation efficiencies of cellulose, hemicellulose and lignin of BSPs. The different normal letters indicate a significant difference in degradation efficiencies of different components with p value at 0.05 level (n = 3)

The gut contents of *C. buqueti* were examined by SEM to investigate the ability of the insect to degrade lignocellulose. The bamboo lignocellulose in the insect was crushed into grain-like particles (Fig. 6b–d) compared to that in the intact bamboo shoot (Fig. 6a), which was similar to that in the termite. Termites use their mandibles to chew through the crop to produce wood particles within a specific size range [31], and Kumar [32] found that termites use their jaws to increase the wood surface area and reduce crystallinity by chewing and grinding.

In addition, we measured the components of lignocellulose (i.e. cellulose, hemicellulose and lignin) in the insects’ faeces. We found that cellulose, hemicellulose and lignin had decreased (Fig. 6e). By examining the structure of the bamboo shoots in the intestine and analysing its composition in the faeces, we observed that lignocellulose was efficiently degraded.

### SEM of components of BSPs and reducing sugar content in culture medium after a 6-day treatment in vitro

**Fig. 7:**
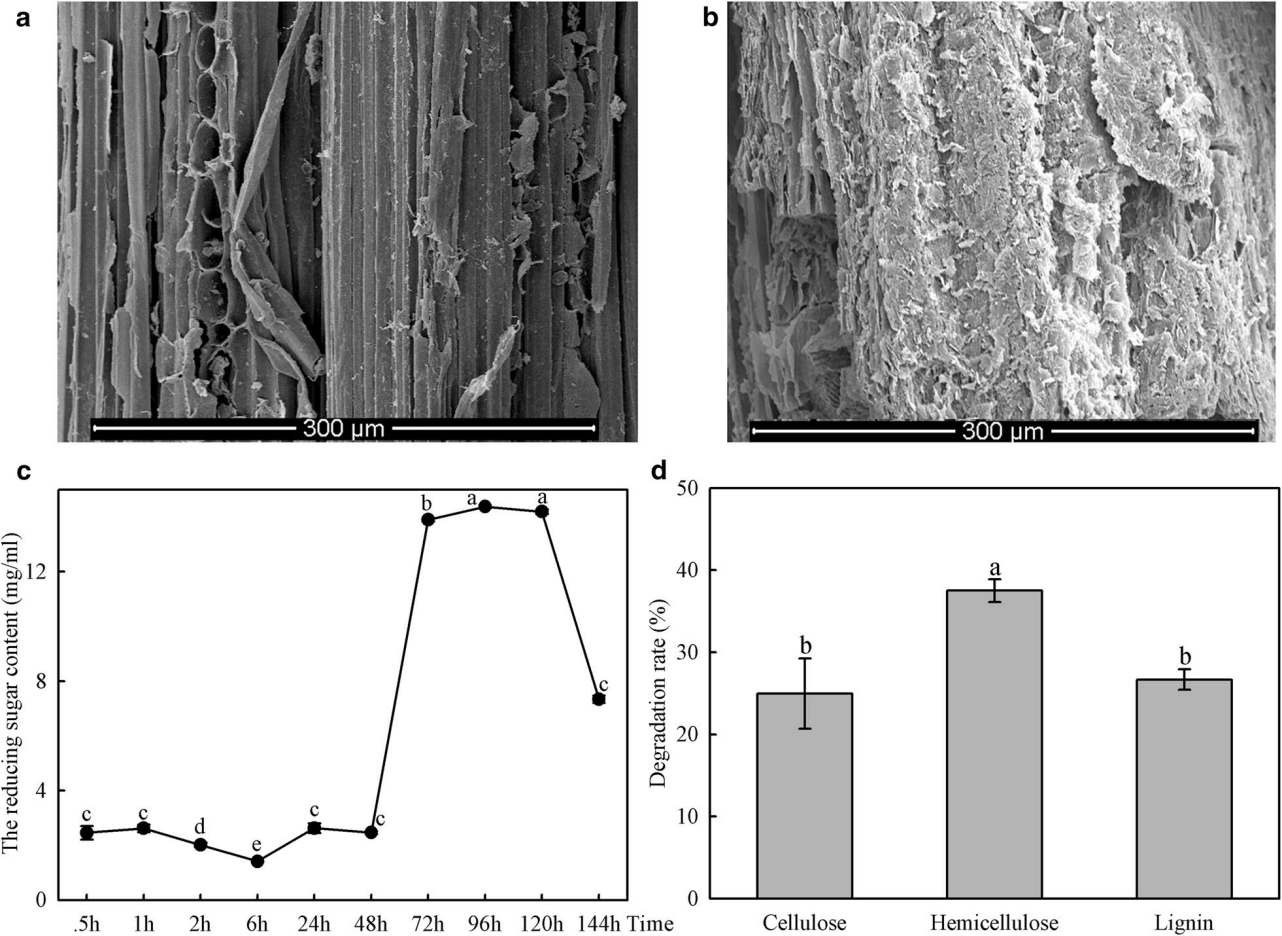
SEM and components of BSPs and reducing sugar content in culture medium after treating for 6 days in vitro. **a** Scanning electron microscopy (SEM) of raw bamboo shoot particles (BSPs); **b** SEM of BSPs after treating for 6 days; **c** amount of reducing sugar in the culture after treating for 6 days; **d** degradation efficiencies of cellulose, hemicellulose and lignin of BSPs after treating for 6 days. The different normal letters indicate a significant difference in degradation efficiencies of different components (*p* = 0.05; n = 3)

To further investigate the catalytic activity of lignocellulolytic enzymes from *C. buqueti*, the crude enzymes were extracted and used to degrade BSPs in vitro for 6 days, after which BSPs were collected for SEM examination. The surface of the untreated BSPs was smooth (Fig. 7a), whereas the dense and ordered structure of BSPs treated with the enzymes was seriously destroyed, and a large number of broken vascular fibres were exposed and were loosely and irregularly arranged; the outer surface layer was shed and a scale-like structure emerged. These results indicated that the treated bamboo fibres changed from a closely connected state to one of separation and exposure. Moreover, the pore structure appeared (Fig. 7b). These results were consistent with those of other reports [33, 34]. The microstructural changes in BSPs before and after treatment showed that their structure was seriously destroyed by treatment with crude enzymes.

Because the reducing sugar in the culture medium was derived mainly from the hydrolysis of cellulose and hemicellulose in BSPs, the reducing sugar content was used to measure the degree of destruction of the lignocellulose structure. The amount of reducing sugar produced by BSPs changed depending on the time frame, as shown in Fig. 7c. Within the first 2 days of culture, the amount of reducing sugar did not increase; however, after 2 days, it began to increase to a maximum value of 14.3798 mg/g on day 4, after which it steadily decreased until day 6. Increases in reducing sugar content indicated the degradation of cellulose and hemicellulose of BSPs by crude enzymes [35]. The delayed decrease could be a result of a growth of microbes in the culture. In addition, we determined the rate of BSP lignocellulose degradation after a 6-day treatment. As shown in Fig. 7d, the degradation efficiencies of cellulose, hemicellulose and lignin from BSPs were 24.98%, 37.52% and 26.67%, respectively.

To overcome the recalcitrance of lignocellulose, pre-treatment is considered as an indispensable procedure that has a pervasive impact on total sugar recovery [36]; therefore, it is necessary to pretreat lignocellulose before hydrolysis to remove lignin and hemicellulose, to reduce the resistance of the raw material to enzymatic hydrolysis and then hydrolyse the cellulose into fermentable sugars [37]. It was observed that lignin and hemicellulose were partially removed by treating BSPs with crude enzymes. Although the cellulose also decreased, these results showed that the enzymes that degraded the lignin and hemicellulose could be expressed in vitro using genetic engineering and applied to pretreat the lignocellulose of bamboo.

To investigate the mechanism by which lignocellulose in BSPs degrade in vitro, we identified lignocellulolytic enzymes, such as endoglucanase, β-glucosidase, xylanase-like enzyme, exoglucanase, laccase and LiP, displayed by the enzyme proteins in the adult insect 0.5, 1, 2, 6, 24, 48, 72, 96, 120 and 144 h after being co-cultured with BSP in vitro (Additional file 7: Figure S2). The results showed that cellulase exhibited a tendency to increase, while changes in the other enzymes were irregular, which could be a result of unstable reaction conditions, such as pH.

### GC–MS analysis of the products of lignin degraded by enzymes co-cultured with BSP in vitro

GC–MS, which has proved to be feasible in detecting products of lignin degradation [38], was used to identify low-molecular weight products from crude enzyme-degraded BSPs. The total ion chromatograms corresponding to compounds extracted from the control and treated samples showed their peak intensities and are listed in Table 1. A number of small molecular aromatic metabolites, such as phenol (RT, 7.24), phenylethanol (RT, 10.64), ethyl guaiacol (RT, 15.06), 4-ethylphenol (RT, 12.42) and p-cresol (RT, 9.90), which are considered as the basic units of the lignin polymer, were detected only in the samples treated by crude enzymes for 6 days (Additional file 8: Figure S3b; Table 1). Lignin can be depolymerised into monomers containing phenolic hydroxyl and benzene, dimers or polyphenols after oxidation, hydrolysis, liquefaction, cracking and enzymatic hydrolysis [39]. This indicates that the BSP lignin was degraded after treatment for 6 days with crude enzymes from the insect.

**Table 1:** Identification of metabolites as trimethylchlorosilane (TMS) derivatives from bamboo shoot particles (BSP) samples

Retention time	Compound	Control^a^	Treated^b^
4.94	Isonicotinic acid	−	+
5.28	1H-Tetrazol-5-amine	−	+
5.99	Pyrrolidine	−	+
6.22	cis-1-Butene	−	+
7.24	Phenol	−	+
9.90	p-Cresol	−	+
10.64	Phenylethyl alcohol	−	+
10.66	Propanedinitrile	−	+
12.42	4-ethyl-Phenol	−	+
15.06	4-ethyl-2-methoxy-Phenol	−	+
20.47	4-hydroxy-Benzaldehyde	−	+

^a^Non-inoculated (control) BSP samples

^b^Crude enzyme-degraded BSP samples

### Application of lignocellulolytic enzymes of *C. buqueti* in bamboo lignocellulose degradation

As an important biomass raw material, the lignocellulose in bamboo is of significant value in energy conversion. The conversion of bamboo lignocellulose into fermentable reducing sugar (i.e. the degradation of bamboo lignocellulose) is an important step in lignocellulose conversion. The degradation of bamboo lignocellulose, including its pre-treatment and enzyme hydrolysis, has focussed mainly on using physical, chemical and commercial enzymes [3–5], and few reports have focussed on degradation by microbes, such as *Coriolus versicolor* [40] and *Galactomyces* sp. CCZU11-1 [41]. In addition, little research has focussed on excavating the enzymes and microbes that are related to the degradation of bamboo lignocellulose.

The digestive system of termites has been described by many scientists as the world’s smallest but most efficient bioreactor [42]. Xie et al. [43] and Geng et al. [9] suggested that a new system of biomass degradation could potentially be established by learning from biological conversion systems using biomimetic technology and combining nature-inspired technology with relative genes and enzymes from termites or other biological systems. In our present study, we report that the intestinal digestive enzymes from the bamboo shoot pest, *C. buqueti*, degraded lignocellulose from bamboo shoots both in vivo and in vitro. In addition, the expression levels of many CAZyme genes were upregulated, including those for cellulase, xylanase and ligninase genes in the transcriptome. Therefore, we proposed a scheme for applying the lignocellulolytic enzymes from *C. buqueti* to degrade bamboo lignocellulose using genetic, enzyme and fermentation engineering to overexpress the lignocellulolytic enzymes genes in vitro and obtain massive amounts of enzymes that could efficiently degrade bamboo lignocellulose and be used for lignocellulose bioconversion.

## Conclusions

The bamboo snout beetle *Cyrtotrachelus buqueti* is an extremely harmful bamboo borer and acts as a ‘natural biomass utilisation system’. However, little work has been done for *C. buqueti* and bamboo degradation. In this study, we determined the degradation efficiency of bamboo lignocellulose, and showed that the degradation efficiencies of cellulose, hemicellulose and lignin in BSPs were 61.82%, 87.65% and 69.05%, respectively, in vivo, and were 24.98%, 37.52% and 26.67%, respectively, in vitro. It indicated the degrading ability in bamboo lignocellulose of the insect. Moreover, the transcriptome showed that, after feeding, many CAZyme gene expressions changed and that lignocellulosic enzyme genes, such as endoglucanase genes (*evgtrinloc27093t1* and *evgtrinloc16407t0*) and laccase genes (*evgtrinloc15173t0* and *evgtrinloc11252t0*), were upregulated, which was in accordance with the enzyme activity. This indicates that lignocellulosic enzymes and those related to bamboo fibre degradation within the insect itself co-degraded bamboo lignocellulosee, which may provide new enzyme resources and ideas for the bioconversion of bamboo lignocellulose.

## Materials and methods

### Insect collection and treatment

Adult *C. buqueti* were collected from Dannan, Sichuan Province (N103°98′, E28°96′) in early August 2017 and were used 3 days after emergence [44]. The insects were starved for 24 h, after which 100 were placed on ten healthy bamboo shoots of similar growth. Each bamboo shoot was surrounded by a 20 × 20 × 50 cm iron cage. After blocking the lower end of the cage with bamboo leaves and other bamboo wastes for 3 h, the entire digestive tracts (including the beak) of the feeding beetles were dissected with distilled water and stored with RNAlater. All samples were immediately frozen in liquid nitrogen and stored at − 80 °C until use. Each sample contained tissues from at least five insects. Each treatment was conducted three times.

### RNA extraction, cDNA library construction and RNA sequencing

Total RNA was extracted from the samples using the DP431 RNAprep Pure Tissue Kit (TianGen Biotechnology, Beijing, China). The yield and purity of the RNA were assessed using 1% agarose gel. An Agilent 2100 Bioanalyzer (Agilent Technologies, Santa Clara, CA, USA) was used to detect RNA integrity. RNA was stored at − 80 °C. Five micrograms of total RNA was used to construct the cDNA library and Illumina sequence using Illumina HiSeq 4000, which was managed by Chengdu Basebiotech Co., Ltd.

### Gene annotation and functional analysis of differentially expressed genes

The following eight databases were used for unigene annotation: Conserved Domain Database (CDD), NCBI protein (Nr), Protein family (PFAM), euKaryotic Orthologous Groups (KOG), Translation from EMBL (TrEMBL), Swiss-Prot, GO, and KEGG Orthology. Blast2GO and KEGG Automatic Annotation Server tools were used for annotation. Trinity was used to assemble the transcriptome [19], and RSEM was used for mapping clean reads [45]. The R package DEseq was used to show significant differences in the expression of the unigenes [46]. The false discovery rate (FDR) q value threshold was set at 0.05, and the fold change of expression was set at 2.0.

GOseq was used for GO analysis based on Wallenius’ non-central hypergeometric distributions [47]. KOBAS 2.0 was used for KEGG enrichment analysis with hypergeometric tests [47]. GO categories and KEGG pathways with an FDR *q* value ≤ 0.05 were considered to be significantly enriched.

### CAZyme family analysis in the transcriptome

To identify the genes involved in lignocellulose degradation, coding sequences (CDS) were analysed using the dbCAN CAZyme annotation algorithm, which gives the hidden Markov model index files of various carbohydrate enzyme domains using hmmscan [48].

### Assays to determine lignocellulolytic enzyme activity

To verify changes in the lignocellulolytic enzymes cellulase, xylanase and ligninase in the intestinal tract after eating bamboo shoots, 30 *C. buqueti* adults that had been denied food for 24 h were placed on bamboo to feed and then collected 0, 0.5, 1 and 2 h after feeding. Their intestines were dissected in an aseptic environment and made into a crude enzyme solution for each of the different feeding periods and then philtre sterilised with a 0.22-μm philtre. The solution was then used to determine lignocellulolytic enzyme activity; assays were conducted five times each. The tissues were then ground, added to 1 mL pH 5.6 phosphate-buffered saline (PBS) extraction buffer, and centrifuged at 13,000×*g* for 10 min at 4 °C. The supernatant was collected, which represented the crude enzyme solution. Each replicate sample contained tissues from at least five insects. The process was conducted five times on each sample.

The assays for endoglucanase (EC 3.2.1.4) and exoglucanase (EC 3.2.1.91) were conducted according to the method of Ghose et al. [49] and the assay for β-glucosidase (EC 3.2.1.21) activity was conducted according to the method of Perry et al. [50]. Briefly, carboxymethyl cellulose (CMC), microcrystalline cellulose (MCC), and salicin were used as substrates to determine endoglucanase, exoglucanase and β-glucosidase, respectively. First, 2 mL 1% CMC, MCC, or salicin was added to a 25-mL test tube and preheated at 50 °C for 2–3 min. Then, 5-mL crude enzyme solution was added and incubated at 50 °C for 30 min, after which 2.5-mL DNS was added and the solution was placed at 100 °C for 5 min to immediately terminate the reaction. Finally, 25 mL of pH 5.6 PBS was added to the solution and the optical density was determined at a wavelength of 540 nm.

LiP-like activity was measured according to the method of Yan et al. [51]. Briefly, veratryl alcohol (VA) was used as a substrate and the reaction was performed in pH 5.6 PBS. The LiP activity was measured by monitoring the oxidation of VA at 310 nm. Laccase-like activity was measured according to the method of Nakagawa et al. [52] in which 2,2′-azino-bis (ABTS) was used as a substrate and the enzyme activity was measured by monitoring ABTS oxidation at 420 nm. All assays were performed five times.

### Tissue RNA extraction and quantitative reverse transcription polymerase chain reaction of lignocellulolytic enzyme genes in the digestive system of *C. buqueti*

Thirty *C. buqueti* adults that were denied food for 24 h were collected 0, 0.5, 1 and 2 h after feeding, and their intestines were rapidly extracted. A DP431RNAprep Pure Tissue Kit (Tiangen Biotech, Beijing, China) was used to extract total RNA from the intestines of the insects. All treatments were conducted three times. The primers used for quantitative reverse transcription polymerase chain reaction (qRT-PCR) analysis are listed in Additional file 9: Table S6. qRT-PCR reactions were conducted using the ABI StepOnePlus™ Real-Time PCR System. All qRT-PCR reactions were conducted three times and analysed using the 2^−ΔΔCT^ method [53].

### Bamboo shoot and faeces collection, determination of the components and SEM

Bamboo shoots 50–100 cm high from *Bambusa emeiensis* were collected from Muchuan (N103°98′, E28°96′) and cut into 5 × 5 cm pieces. The pieces were divided into two parts: one was directly used as food for *C. buqueti*; the other was strained through a 40-mesh sieve, dried at 65 °C for 72 h and stored at 4 °C.

The insects were fed bamboo shoot pieces that measured 30 cm in diameter, placed in a high 10-cm-diameter plastic box and reared in the laboratory at 25 °C ± 1 °C and 70% ± 10% relative humidity with a 12L:12D photoperiod [44]. Faeces were collected each day from the inner surface of the plastic box, immediately placed in a cryopreservation tube and stored at − 80 °C.

BSPs and faeces were examined by SEM. The surface morphology of the samples was observed by SEM (Hitachi 3400N, Japan) and the samples were sprayed with gold to a thickness of approximately 10 nm using an E-1010 sputtering film coating machine (Japan) before imaging. The operating current and voltage of the SEM were 81 mA and 10 kV, respectively.

### Assay of crude enzymes in vitro

To prepare BSPs for assay, the shoots were dried to a uniform weight at 65 °C, then crushed into particles using a pulverising machine and strained through a 40-mm-mesh sieve.

The tissues of 50 *C. buqueti* adults were used for the process of extracting the crude enzyme proteins. The tissues were ground into 10 mL pH 5.6 PBS extraction buffer, the crude extract was then centrifuged at 13,000×*g* for 10 min at 4 °C and the supernatant collected and then philtre sterilised with a 0.22-μm philtre. Each replicate sample contained tissues from 10 insects, and the process was conducted five times on each sample.

For the assay, 10-mL 80 g/L tetracycline solution was prepared. As shown in Table 2, the samples were added into a 250-mL conical flask and incubated under constant-temperature shock at 37 °C and 150 rpm for 6 d. The reaction products were inactivated at 100 °C for 30 min, centrifuged at 13,000 rpm for 10 min, and the supernatant collected and dried at 65 °C to a uniform weight. The dried deposit was weighed and the cellulose, hemicellulose and lignin were determined and used for SEM. The supernatant was used for determining the reducing sugar and low-molecular weight products.

**Table 2:** Design and determination methods of crude enzyme degradation of bamboo shoot particles (BSPs) in vitro

	Experimental group	Control group	Incubation time	Temperature
Experimental design	2 mL crude enzyme + 98 mL PH 5.6 PBS buffer + 10 μL tetracycline + 5 g BSPs	100 ml PH 5.6 PBS buffer + 10 μL tetracycline + 5 g BSPs	6 days	37 °C
Determination index and methods	Low-molecular weight products	Lignocellulose	Surface structure of BSPs	Reducing sugar
	GC–MS (Raj et al. 2007c)	Van Soest method (1991)	SEM	DNS (Miller et al. 1959)

#### Determination of reducing sugar

Reducing sugar was identified using 3,5-dinitrosalicylic acid [54]. Firstly, preparation of 3,5-dinitrosalicylic acid solution with 6.3 g 3,5-dinitrosalicylic acid, 262-ml 2 mol/L NaOH, 182-g Seignette salt, 5-g phenol and 5-g sodium sulphite was conducted. Secondly, 0-, 0.2-, 0.4-, 0.6-, 0.8- and 1.0-mL glucose standard solution (1 mg/mL) was used to determine the glucose standard curve. Finally, 1.0-mL supernatant was added to the 15-mL calibration tube with 2.0 mL of DNS reagent and boiled for 2 min, then cooled and the determined at 540-nm wavelength. The reducing sugar content was then calculated according to the glucose standard curve.

#### Determination of cellulose, hemicellulose and lignin

The cellulose, hemicellulose, and lignin contents of the dried BSPs were determined using the Van Soest method [55] and the following formulas:

$${\text{Hemicellulose content}}\, = \,{\text{neutral detergent fibre }}\left( {\text{NDF}} \right)\, - \,{\text{acid detergent fibre }}\left( {\text{ADF}} \right)$$

$${\text{Cellulose content}}\, = \,{\text{ADF}}\, - \,{\text{acid detergent lignin }}\left( {\text{ADL}} \right)$$

$${\text{Lignin content}}\, = \,{\text{ADL}}\, - \,{\text{ash content}};$$

#### Cellulose, hemicellulose and lignin degradation efficiencies

The following equations were used for calculating degradation efficiency:

$${\text{Degradation efficiency of cellulose }} = \, \left( { 1- \frac{\text{The mass of cellulose in deposit }}{\text{The mass of cellulose in raw material}}} \right) \times { 1}00\%$$

$${\text{Degradation efficiency of hemicellulose }} = \, \left( { 1- \frac{\text{The mass of hemicellulose in deposit }}{\text{The mass of hemicellulose in raw material}}} \right) \times { 1}00\%$$

$${\text{Degradation efficiency of lignin }} = \left( { 1- \frac{\text{The mass of lignin in deposit }}{\text{The mass of lignin in raw material}}} \right) \times { 1}00\%$$

#### Determination of the lignocellulolytic enzymes activities

From the co-cultures, 2-mL samples were withdrawn at 0.5, 1, 2, 6, 24, 48, 72, 96, 120 and 144 h for assays of lignocellulolytic enzyme activity. The determination method was same as that used above.

#### Gas chromatography–mass spectrometry (GC–MS)

Cultures of BSP treated with crude enzymes were collected on day 6 and centrifuged at 8000 rpm for 15 min to remove biomass. The supernatants were collected and treated using the method of Raj et al. [38]. In brief, the supernatants were extracted three times using equal volumes of dichloromethane. The extracted liquor was collected, dewatered over anhydrous Na_2_SO_4_, filtered through philtre paper and concentrated to ~ 1 mL using a rotary vacuum evaporator. Then, 100-µL dioxane and 10-µL pyridine were added to the sample followed by silylation with 50-µL trimethyl silyl (N,O-bis [trimethylsilyl] trifluoroacetamide [BSTFA]/trimethylchlorosilane [TMS] = 99/1 [v/v]). Gas chromatography–mass spectrometry (GC–MS) was used to analyse the silylated compounds according to the procedure of Chen et al. [56]. The TMS derivatives from crude enzyme degradation were identified by comparing their mass spectra with that of the NIST library available on the equipment.

### Statistical analysis

Statistical analyses were performed using SPSS 19.0 (IBM Corporation, Inc., Armonk, NY, USA). Descriptive data were expressed as the mean ± standard error of mean (SEM). A Student’s *t* test was used to compare the means from two groups. Intergroup comparisons of more than two groups were conducted using analysis of variance. A *p* value of less than 0.05 indicated a statistically significant difference.

## Additional files

**Additional file 1: Table S1.** Number and length of transcripts and unigenes and unigenes annotated in different databases.

**Additional file 2: Table S2.** Percentage numbers of the abundant annotated species.

**Additional file 3: Fig. S1.** The number of CAZymes in the transcriptome. GHs: glycoside hydrolases, GTs: glycosyltransferases, CEs: carbohydrate esterases, CBMs: carbohydrate-binding domains, PLs: polysaccharide lyases, AAs: auxiliary activities, CAZyme: carbohydrate-active enzymes.

**Additional file 4: Table S3.** Unigenes annotated in CAZyme.

**Additional file 5: Table S4.** The CAZyme classification at the family level.

**Additional file 6: Table S5.** The annotation of genes in CAZyme in the transcriptome.

**Additional file 7: Fig. S2.** The lignocellulolytic enzyme activities of *C. buqueti* displayed by enzymes of the adults 0.5, 1, 2, 6, 24, 48, 72, 96, 120 and 144 h after being co-cultured with BSP in vitro.

**Additional file 8: Fig. S3.** Total ion chromatograms of dichloromethane extract analysed as trimethylchlorosilane derivatives from control (a) and treatment (b) in vitro.

**Additional file 9: Table S6.** The primer sequence of qRT-PCR.

## Abbreviations

*C. Buqueti*: *Cyrtotrachelus buqueti*
NBUS: natural biomass utilisation systems
RT-PCR: reverse transcription polymerase chain reaction
GHs: glycoside hydrolases
GTs: glycosyltransferases
CEs: carbohydrate esterases
CBMs: carbohydrate-binding domains
PLs: polysaccharide lyases
AAs: auxiliary activities
CAZyme: carbohydrate-active enzymes
MnP: manganese peroxidases
LiP: lignin peroxidase
Lac: laccase
cDNA: complementary deoxyribonucleic acid
RNA: ribonucleic acid
SEM: scanning electron microscope
TPM: Transcripts Per Million
GO: gene ontology
KEGG: the database of Kyoto encyclopedia of genes and genomes
FPKM: fragments per kilobase of transcript, per million fragments sequenced
ABTS: [2,2′-Azino-bis (3-ethylbenzothiazoline-6-sulfonic acid)]
