# Supplementary figures and images for: Degradation of bamboo lignocellulose by bamboo snout beetle Cyrtotrachelus buqueti in vivo and vitro: efficiency and mechanism
Source: Biotechnol Biofuels. 2019 Apr 1;12:75. doi: 10.1186/s13068-019-1406-y (PMC6442404; doi:10.1186/s13068-019-1406-y)

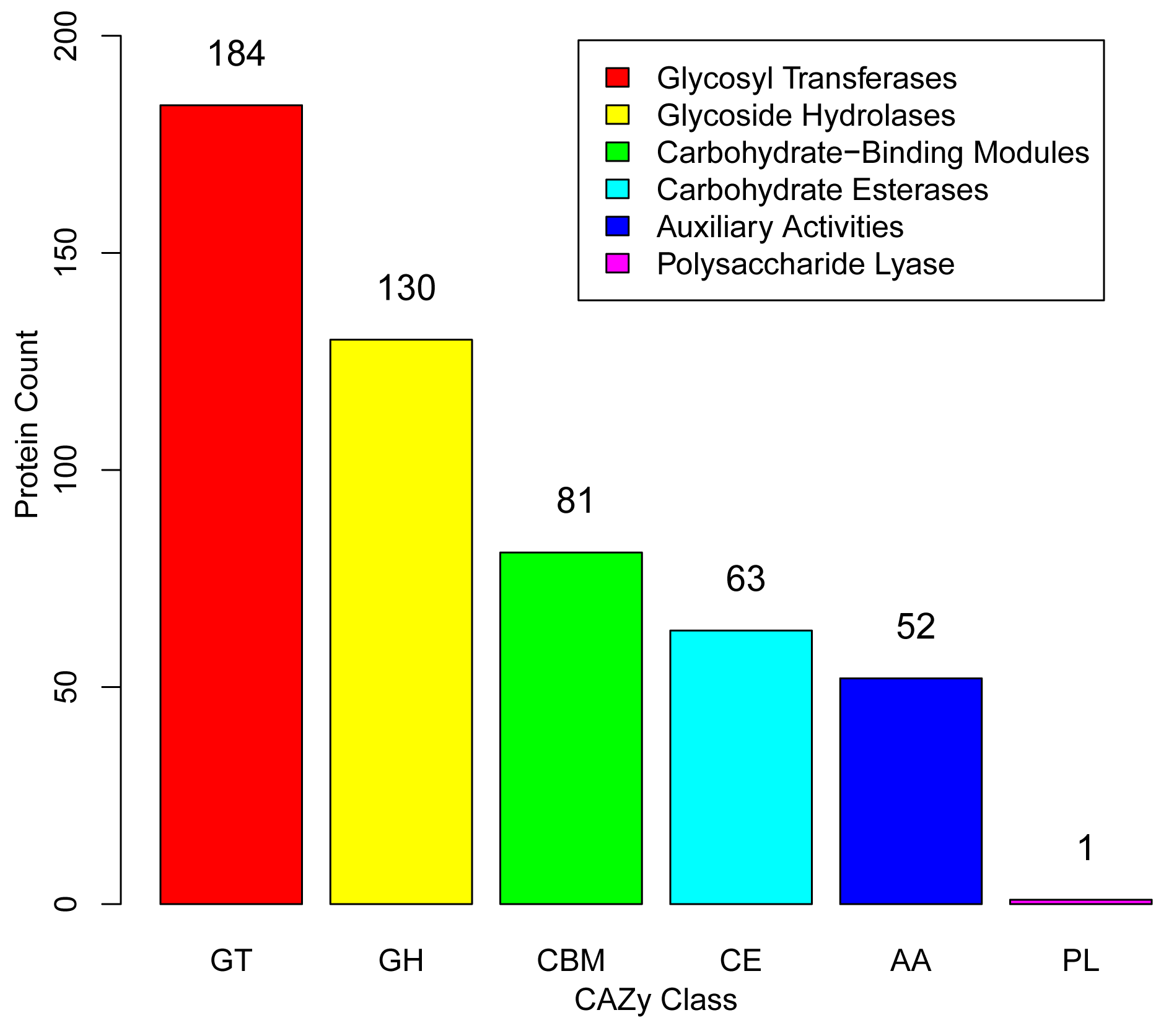

Supplement: Supplementary file 3 — Additional file 3: Fig. S1. The number of CAZymes in the transcriptome. GHs: glycoside hydrolases, GTs: glycosyltransferases, CEs: carbohydrate esterases, CBMs: carbohydrate-binding domains, PLs: polysaccharide lyases, AAs: auxiliary activities, CAZyme: carbohydrate-active enzymes. [file 13068_2019_1406_MOESM3_ESM.tif]

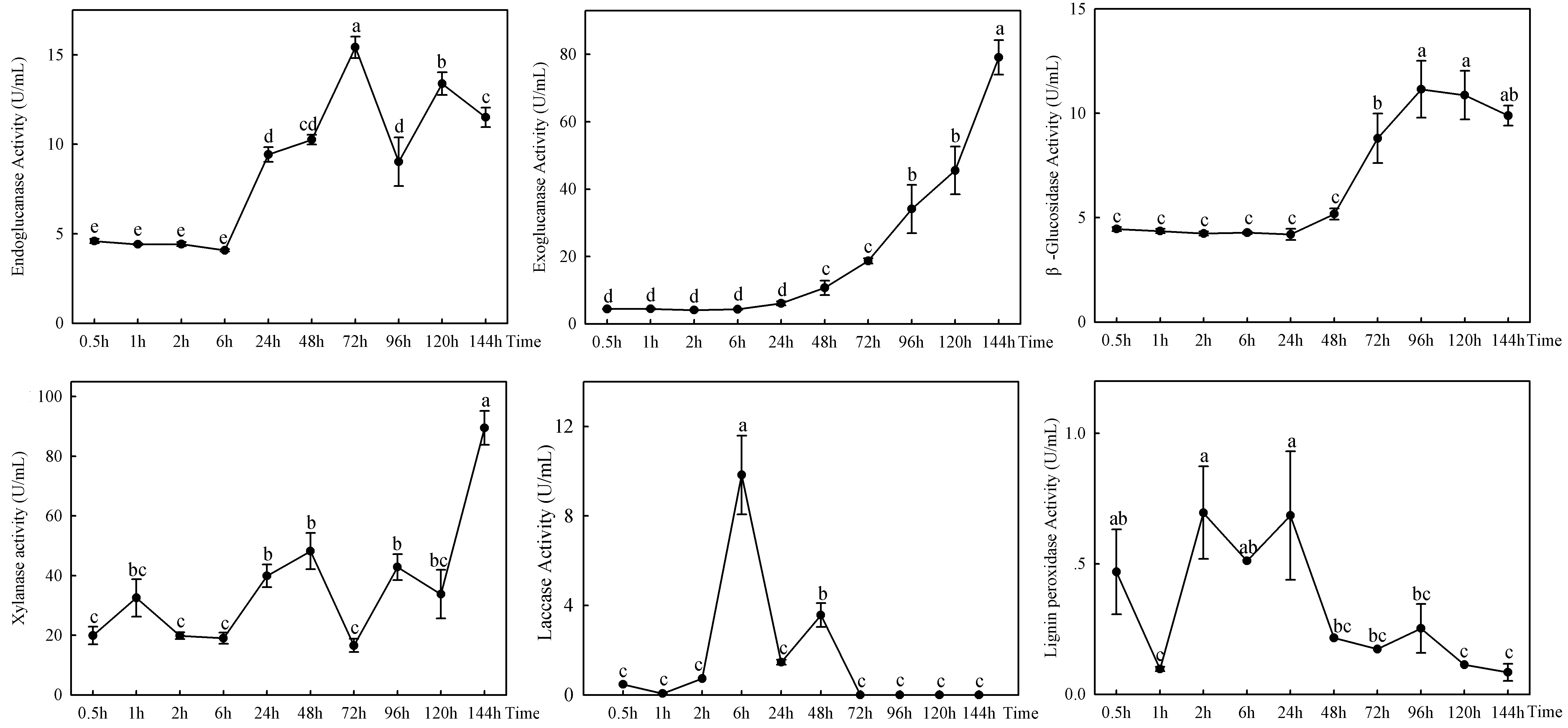

Supplement: Supplementary file 7 — Additional file 7: Fig. S2. The lignocellulolytic enzyme activities of C. buqueti displayed by enzymes of the adults 0.5, 1, 2, 6, 24, 48, 72, 96, 120 and 144 h after being co-cultured with BSP in vitro. [file 13068_2019_1406_MOESM7_ESM.tif]

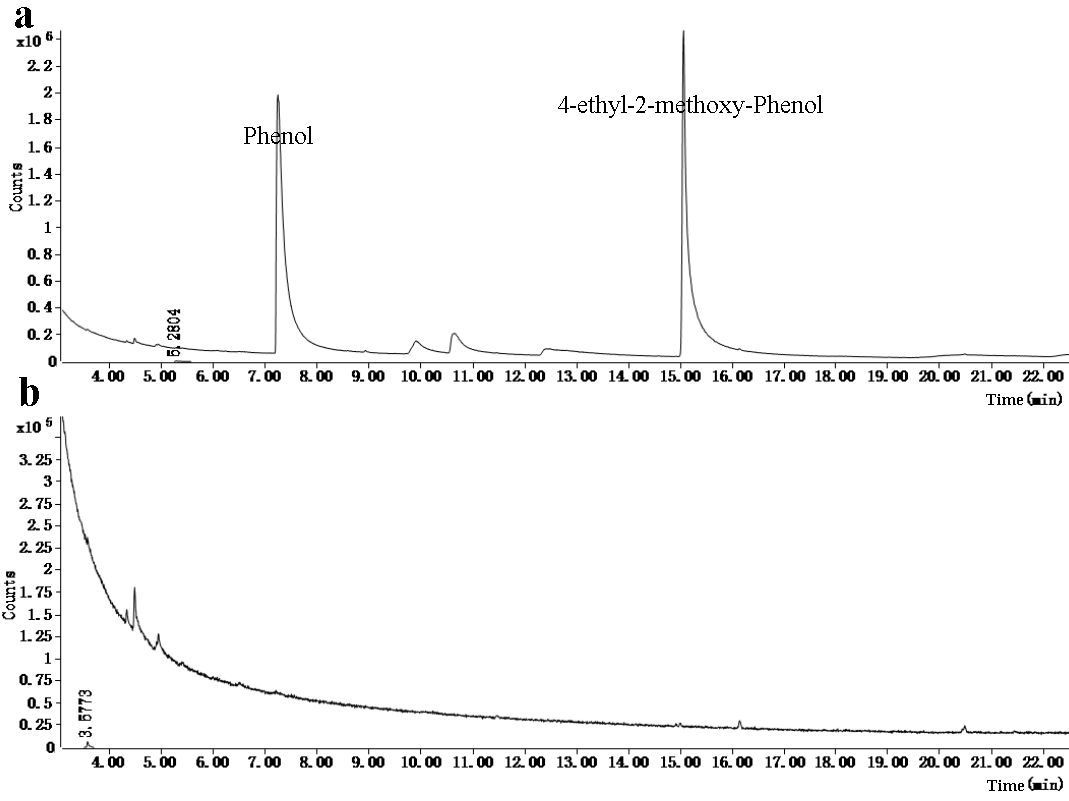

Supplement: Supplementary file 8 — Additional file 8: Fig. S3. Total ion chromatograms of dichloromethane extract analysed as trimethylchlorosilane derivatives from control (a) and treatment (b) in vitro. [file 13068_2019_1406_MOESM8_ESM.tif]
